# Socioeconomic Status Correlates with the Prevalence of Advanced Coronary Artery Disease in the United States

**DOI:** 10.1371/journal.pone.0046314

**Published:** 2012-09-25

**Authors:** Bronislava Bashinskaya, Brian V. Nahed, Brian P. Walcott, Jean-Valery C. E. Coumans, Oyere K. Onuma

**Affiliations:** 1 Massachusetts General Hospital and Department of Medicine (Cardiology Division), Harvard Medical School, Boston, Massachusetts, United States of America; 2 Boston University, Boston, Massachusetts, United States of America; 3 Massachusetts General Hospital and Department of Surgery (Neurosurgery Division), Harvard Medical School, Boston, Massachusetts, United States of America; Sapienza University of Rome, Italy

## Abstract

**Background:**

Increasingly studies have identified socioeconomic factors adversely affecting healthcare outcomes for a multitude of diseases. To date, however, there has not been a study correlating socioeconomic details from nationwide databases on the prevalence of advanced coronary artery disease. We seek to identify whether socioeconomic factors contribute to advanced coronary artery disease prevalence in the United States.

**Methods and Findings:**

State specific prevalence data was queried form the United States Nationwide Inpatient Sample for 2009. Patients undergoing percutaneous coronary angioplasty and coronary artery bypass graft were identified as principal procedures. Non-cardiac related procedures, lung lobectomy and hip replacement (partial and total) were identified and used as control groups. Information regarding prevalence was then merged with data from the Behavioral Risk Factor Surveillance System, the largest, on-going telephone health survey system tracking health conditions and risk behaviors in the United States. Pearson's correlation coefficient was calculated for individual socioeconomic variables including employment status, level of education, and household income. Household income and education level were inversely correlated with the prevalence of percutaneous coronary angioplasty (−0.717; −0.787) and coronary artery bypass graft surgery (−0.541; −0.618). This phenomenon was not seen in the non-cardiac procedure control groups. In multiple linear regression analysis, socioeconomic factors were significant predictors of coronary artery bypass graft and percutaneous transluminal coronary angioplasty (p<0.001 and p = 0.005, respectively).

**Conclusions:**

Socioeconomic status is related to the prevalence of advanced coronary artery disease as measured by the prevalence of percutaneous coronary angioplasty and coronary artery bypass graft surgery.

## Introduction

Despite preventive measures and aggressive therapy, coronary artery disease (CAD) is responsible for one out of every six deaths in the United States [1]. An estimated 785,000 individuals have a new myocardial infarction every year and more than half have a recurrent attack [1]. It is well known that a multitude of modifiable risk factors contribute to coronary artery disease. These factors include cholesterol levels, smoking status, hypertension, obesity, psychosocial status, consumption of fruits, vegetables, alcohol, physical activity, smoking status, and many more [2] [3]. Modification of these risk factors, presumably as a result of preventive outpatient care, can have dramatic effects on the primary prevention of CAD. This has been seen in studies on the effects of cholesterol modification with HMG-CoA Reductase inhibitors [4]–[6] in addition to the non-pharmacologic effects of diet, exercise, and smoking abstinence [7].

Healthcare in the United States is not universally equitable leading to disparities in access to preventive and primary care. Modifiable CAD risk factors such as cigarette smoking [8], hyperlipidemia [9], [10], and diabetes [1], [11] have been shown to be disproportionately linked to socioeconomic factors. A study examining these risk factors specifically as they relate to cardiovascular disease has determined that while longitudinal improvements are being made, not all sub-populations in society are equally benefiting. Disparities related to education and income based sub-populations associated with these risk factors are increasingly worse [12], [13]. For example, African-American adults have among the highest rates of hypertension in the world (>43%) [1]. These ultimately summate into differences in cardiovascular disease that are recognizable at a geographic (state) level [14].

Socioeconomic factors influence the prevalence of well-established CAD risk factors, and likely influence the prevalence of advanced CAD. Using a disease prevalence approach rather than risk factor analysis, we aim to identify the significance of distinct populations based on income, education level, and employment status as they relate to advanced CAD. This is of significant importance given the recent focus on improvement with healthcare utilization and quality.

## Methods

This study was determined exempt from the Massachusetts General Hospital Institutional Review Board given the de-identified nature of the dataset. To protect confidentiality of patients, the dataset provided suppressed reporting when values were based on 10 or fewer discharges or when fewer than two hospitals in the state were reporting. Survey data was previously obtained via telephone interview from adults 18 years or older who gave verbal consent for de-identified participation. Only one adult was interviewed per household and participants were not compensated.

State specific prevalence data from the US Nationwide Inpatient Sample was queried from the most recent available year, 2009. Weighted national estimates were provided from the Agency for Healthcare Research and Quality (AHRQ), Healthcare Cost and Utilization Project's Nationwide Inpatient Sample (NIS) for 2009, based on data collected by individual states and provided to the AHRQ. The total number of weighted discharges in the U.S. is based on the NIS total of  = 39,434,956. Statistics based on estimates with a relative standard error (standard error/weighted estimate) greater than 0.30 were excluded. Statistics were only based on hospitals that meet the definition of “community hospital” - nonfederal, short-term, general and other specialty hospitals, including public hospitals and academic medical centers. Excluded from the analysis were federal, rehabilitation, and psychiatric hospitals, as well as alcoholism/chemical dependency treatment facilities.

The principal procedure was defined as the definitive treatment during the hospital admission (not diagnostic or exploratory). The unit of identification was the discharge: if a particular procedure occurred multiple times during the same discharge, it was only counted once. State-specific prevalence data was then used for further analysis.

Coronary artery bypass graft (CABG) and percutaneous transluminal coronary angioplasty (PTCA) were identified as principal procedures using clinical classifications software (CCS) of ICD-9-CM codes 44 and 45, respectively [15]. Additional procedures, including lung resection and hip replacement (partial and total) were identified as control groups. [Table 1] Information regarding prevalence and in-hospital mortality for each procedure was merged with data from the Behavioral Risk Factor Surveillance System (BRFSS), the largest, on-going telephone health survey system tracking health conditions and risk behaviors in the United States. Statistical analysis of the distribution of results was performed with Prism 5 and InStat for Mac (GraphPad Software, Inc). First, a univariate analysis was performed to evaluate outcomes (population-adjusted procedure prevalence) and the individual socioeconomic variables including employment status, level of education, and household income. A correlation coefficient was calculated independently, without considering the other variables. For the variable of “unemployed for greater than one year”, homemakers, students, retired persons, self-employed persons, and those unable to work were excluded. Value ranges of 0–0.09, 0.1–0.3, 0.31–0.5, and 0.51–1.0 were considered to have no, small, medium, and strong correlations, respectively. Next, a multiple linear regression model was constructed to account for all three socioeconomic variables and the population-adjusted prevalence for each procedure. Significance was pre-defined at p<0.05.

**Table 1:** ICD-9-CM codes grouped according to clinical classifications software.

CCS Code	Procedure	ICD-9-CM Codes
36	Lung resection; lobectomy or pneumonectomy	3220 3221 3222 3223 3224 3225 3226 3227 3229 323 3230 3239 324 3241 3249 325 3250 3259
44	Coronary artery bypass graft (CABG)	3610 3611 3612 3613 3614 3615 3616 3617 3619 362 363 3631 3632 3633 3634 3639
45	Percutaneous transluminal coronary angioplasty (PTCA)	0066 1755 3601 3602 3605
153	Hip replacement; total and partial	0070 0071 0072 0073 0074 0075 0076 0077 0085 0086 0087 8151 8152 8153 8169

## Results

**Figure 1:**
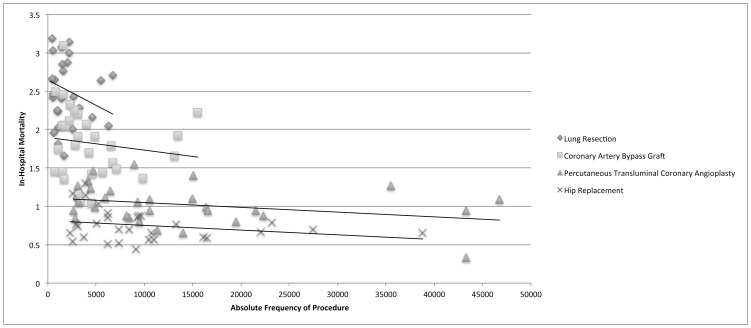
In-hospital morality decreases with increasing absolute frequency of a procedure.

Thirty states provided adequate data for interpretation. [Table 2] There was a small to medium negative correlation with prevalence of procedure and in-hospital mortality for all procedures in a state-by-state analysis (Pearson's correlation coefficient (r) range −0.137 to −0.303). [Figure 1] That is, as the absolute frequency of each procedure increased, the in-hospital mortality decreased.

**Table 2:** States with data available for analysis.

Arizona
Arkansas
California
Colorado
Florida
Illinois
Iowa
Kansas
Kentucky
Maine
Maryland
Massachusetts
Michigan
Minnesota
Missouri
Nebraska
Nevada
New Jersey
New Mexico
New York
North Carolina
Oklahoma
Oregon
South Carolina
Tennessee
Texas
Utah
Washington
West Virginia
Wisconsin

For lung resection (CCS 36), there were a total of 58,176 discharges available for analysis with a mean in-hospital mortality of 2.51%. There was no correlation with the population adjusted prevalence and the percentage of adults unemployed for greater than one year (r = 0.045). However, there were small negative correlations with both household income greater than $ 50,000 USD and having more than a high school education and the population adjusted prevalence (r = −0.130 and −0.188, respectively).

**Figure 2:**
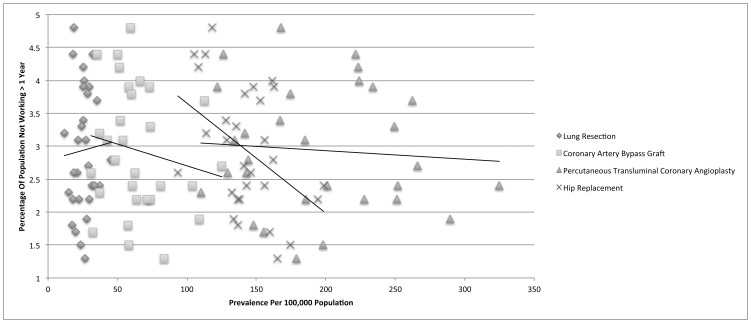
Unemployment for greater than one year is not associated with advanced coronary artery disease.

**Figure 3:**
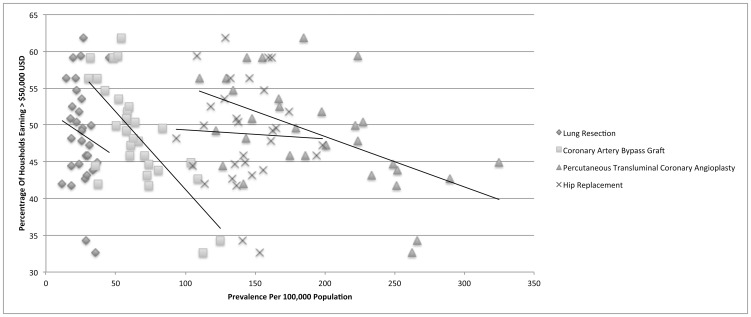
Income is strongly correlated with the prevelance of advanced coronary artery disease.

**Figure 4:**
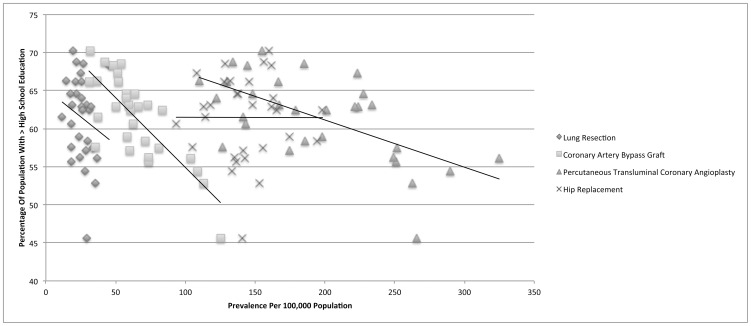
Education level is strongly correlated with the prevalence of advanced coronary artery disease.

For coronary artery bypass graft (CCS 44), there were a total of 135,139 discharges available for analysis with a mean in-hospital mortality of 1.82%. There was a small negative correlation with the population adjusted prevalence and the percentage of adults unemployed for greater than one year (r = −0.167). Importantly, there were strong negative correlations with both household income greater than $ 50,000 USD and having more than a high school education and the population adjusted prevalence (r = −0.717 and −0.787, respectively). [Figures 2, 3, 4]

For percutaneous transluminal coronary angioplasty (CCS 45), there were a total of 428,186 discharges available for analysis with a mean in-hospital mortality of 1.02%. There was no correlation with the population adjusted prevalence and the percentage of adults unemployed for greater than one year (r = 0.076). There were strong negative correlations with both household income greater than $ 50,000 USD and having more than a high school education and the population adjusted prevalence (r = −0.541 and −0.618, respectively).

For hip replacement (CCS 153), there were a total of 303,741 discharges available for analysis with a mean in-hospital mortality of 0.75%. There was a medium negative correlation with the population adjusted prevalence and the percentage of adults unemployed for greater than one year (r = −0.431). However, there was no discernable correlation with either household income greater than $ 50,000 USD or having more than a high school education and the population adjusted prevalence (r = −0.042 and −0.002, respectively). A summary of the Pearson's correlation coefficient calculations for all procedures is provided in Table 3.

**Table 3:** Relationship between individual socioeconomic variables and the prevalence of different procedures.

	Employment	Education	Income
**Lung resection; lobectomy or pneumonectomy**	0.045	−0.130	−0.188
**Coronary artery bypass graft (CABG)**	−0.167	**−0.717** **	**−0.787** **
**Percutaneous transluminal coronary angioplasty (PTCA)**	0.076	**−0.541** **	**−0.618** **
**Hip replacement; total and partial**	−0.431	−0.042	−0.002

** Strong correlation, Pearson's correlation coefficient.

A correlation matrix was established for various procedures (column 1) and socioeconomic factors (columns 2–4). A Pearson's correlation coefficient was established for each relationship. A negative value indicates a negative correlation. Value ranges of 0–0.09, 0.1–0.3, 0.31–0.5, and 0.51–1.0 were considered to have no, small, medium, and strong correlations, respectively. CABG and PTCA had a strong negative correlation with both education and income.

Socioeconomic Factors Key.

Employment = unemployed for greater than one year.

Education = having more than a high school education.

Income = household income greater than $ 50,000 USD.

Finally, a multiple linear regression analysis was performed with all three socioeconomic variables for each procedure. [Table 4] Socioeconomic factors were significant predictors of coronary artery bypass graft and percutaneous transluminal coronary angioplasty (p<0.001 and p = 0.005 for overall model, respectively).

**Table 4:** Multiple linear regression analysis of socioeconomic factors.

Procedure	Variable	Regression Coefficient	95% Confidence Interval	Goodness of Model Fit(R-squared)	Overall Model Significance(P value)
Lung resection; lobectomy or pneumonectomy	Education	−0.491	−1.63, 0.64	0.047	0.732
	Income	0.218	−0.68, 1.12		
	Employment	0.567	−2.49, 3.62		
Coronary artery bypass graft (CABG)	Education	−2.774	−5.10, −0.45	0.641	**<0.001** **
	Income	−0.509	−2.35, 1.33		
	Employment	−3.708	−9.96, 2.54		
Percutaneous transluminal coronary angioplasty (PTCA)	Education	−6.331	−13.39, 0.73	0.390	**0.005** **
	Income	0.169	−5.42, 5.76		
	Employment	−2.708	−21.71, 16.29		
Hip replacement; total and partial	Education	1.711	−1.79, 5.21	0.221	0.086
	Income	−1.450	−4.22, 1.32		
	Employment	−12.201	−21.62, −2.79		

** Significant.

A multiple linear regression analysis was performed for each procedure (column 1) and three socioeconomic factors (column 2). Individual regression coefficients are identified (column 3), along with their respective 95% confidence intervals (column 4). The goodness of model fit (column 5) is the percent of the variation explained by the model. The P value (column 6) represents the significance of each regression model as a whole, incorporating education, income, and employment as variables. This model was significant in describing the relationship of the three socioeconomic variables and the prevalence of CABG and PTCA. No causal mechanism can be identified with any regression analysis technique.

## Discussion

Some disease processes can be analyzed with sufficient sensitivity and specificity on a large scale by identifying procedures related to their advanced stage. For example, early diagnosis and aggressive treatment of *Helicobacter pylori* infection has led to decreased incidence of advanced gastrointestinal ulcer hemorrhage or bowel perforation, and as result fewer surgical procedures related to this diagnosis [16]. Alternatively, improved diagnosis and aggressive treatment may lead to increased frequency of procedures secondary to efficacy as with surgical procedures for ischemic stroke [17].

In this manuscript, we used well-established surrogates for advanced coronary artery disease, coronary artery bypass graft and percutaneous transluminal coronary angioplasty, and investigated their relationship with socioeconomic factors [18]–[20].

The number of cardiac revascularization procedures was inversely related to highest education levels of the patients. This coincides with an established body of evidence that the highest formal education level corresponds to known risk factors for heart disease, such as obesity [21], diabetes [22], and hypertension [23]. Education level is also an established and well known correlate of non-cardiac related conditions, such as cancer [24], rheumatoid arthritis [25], cerebrovascular disease [23], and back pain [26]. Education level is an important marker of socioeconomic status not only because it describes the educational attainment that may confer a better understanding and self-management of preventative health measures, but it also indirectly relates to earning potential (household income) and employment status that can both influence ones ability to obtain routine healthcare.

Coronary artery disease often results from a culmination of multiple patient-centered factors such as diet and exercise [27]. Lifestyle choices such as diet and activity level influence cardiovascular disease risk factors such as hypertension and diabetes mellitus and in turn the development of coronary atherosclerosis [28]–[32]. Education, both in the classroom setting and via a healthcare provider are likely to influence patient compliance with healthy lifestyle choices as they relate to cardiovascular disease prevention. This provides some explanation to the strong negative correlation of education and the decreased prevalence of coronary artery disease.

Another finding of this study was that income levels correlate with the prevalence of advanced coronary artery disease. Household income levels have previously been associated with health insurance status, medical care use, health, and employment [33]. In a complex interplay of these factors, household income provides a summative metric to compare groups. We chose a value much higher than the defined poverty level in an effort to compensate for the anticipated costs of a balanced diet and healthcare coverage. Even at the generous mark of $ 50,000 USD, a strong correlation was present. It should be noted that no accounting could be made for household size in relationship to income level, as this data does not exist in the accessed databases.

Additional factors that likely influence the development of advanced cardiovascular disease include biological differences in certain ethnic and gender groups not accounted for in this study, such as factors that alter the interaction between prothrombotic factors and atherosclerosis [13]. It has been shown that living in a disadvantaged neighborhood is a risk factor for coronary heart disease, even after controlling for income and education [34].

The strength of this study includes the use of two well-established national databases that encompass hundreds of thousands of people, enabling a robust comparison between study and control groups. Limitations include the geographic reporting at the state level that make regional differences at the neighborhood or even city level difficult to account for. There also exist state-to-state differences in practice patterns between CABG and PTCA. In one of the largest studies of regional discrepancies in treatment modalities for acute myocardial infarction cardiac revascularization, state specific CABG rates varied from 9.3% to 13.1% [35]. The state specific rates for PTCA associated with acute myocardial intervention varied much more widely, ranging from 16.8 to 36.0% [35]. State specific factors may influence whether one is more likely to undergo CABG or PCTA for acute myocardial infarction, however no data currently exist regarding the prevalence of these procedures in all settings.

Inherent to national databases is the potential for geographical biases with respect to aggressiveness of intervention. For example, a patient with multiple medical comorbidities in poor clinical condition may be considered a candidate for CABG in one state but perhaps not in another. These differences are difficult to quantify from a population-based standpoint and are best addressed with prospective, intention to treat analysis. These databases also lack information on the degree of severity of the coronary artery blockage and do not capture clinical data points such as time interval from symptom onset to treatment. All data recorded in national databases are subject to various coding anomalies. We attempted to eliminate this bias by focusing exclusively on the principal diagnosis (that is the major determinant of reimbursement rates) with the assistance of CCS grouping that systematically and compressively identifies key ICD-9-CM procedure codes. Additionally, not all states participate in the BRFSS, limiting the analysis to the data of 30 states. This study is expected to generally underestimate differences in health status, as the amount of undiagnosed disease in those without *any* access to care is impossible to report.

## Conclusions

Socioeconomic factors are associated with the prevalence of advanced coronary artery disease, as defined by the geographic prevalence of percutaneous transluminal coronary angioplasty and coronary artery bypass graft surgery. Additional investigation is needed to better define and mitigate the role of socioeconomic factors on the burden of coronary artery disease.
